# En bloc removal of a giant renal calculus in a single sitting via transperitoneal laparoscopic pyelolithotomy: a safe and effective minimally invasive alternative

**DOI:** 10.1093/jscr/rjag750

**Published:** 2026-08-31

**Authors:** Dharmendra Kumar Pipal, Gaurav Gupta, Swati Prasad, L Rajesh Kannan, Alan Philips, B Nikhil, Shashikant Sai, Raja Murtaza Aaqib, Vikas Prasad, Pearl Yadav, Divya Mishra, Twvisham Srivastav, Nischit B Murgod

**Affiliations:** Department of General Surgery, All India Institute of Medical Sciences, Kunraghat, Gorakhpur 273008, India; Department of General Surgery, All India Institute of Medical Sciences, Kunraghat, Gorakhpur 273008, India; Department of General Surgery, All India Institute of Medical Sciences, Kunraghat, Gorakhpur 273008, India; Department of General Surgery, All India Institute of Medical Sciences, Kunraghat, Gorakhpur 273008, India; Department of General Surgery, All India Institute of Medical Sciences, Kunraghat, Gorakhpur 273008, India; Department of General Surgery, All India Institute of Medical Sciences, Kunraghat, Gorakhpur 273008, India; Department of General Surgery, All India Institute of Medical Sciences, Kunraghat, Gorakhpur 273008, India; Department of General Surgery, All India Institute of Medical Sciences, Kunraghat, Gorakhpur 273008, India; Department of General Surgery, All India Institute of Medical Sciences, Kunraghat, Gorakhpur 273008, India; Department of General Surgery, All India Institute of Medical Sciences, Kunraghat, Gorakhpur 273008, India; Department of General Surgery, All India Institute of Medical Sciences, Kunraghat, Gorakhpur 273008, India; Department of General Surgery, All India Institute of Medical Sciences, Kunraghat, Gorakhpur 273008, India; Department of General Surgery, All India Institute of Medical Sciences, Kunraghat, Gorakhpur 273008, India

**Keywords:** laparoscopy, pyelolithotomy, giant renal calculus, kidney stones, minimally invasive surgical procedures, percutaneous nephrolithotomy

## Abstract

Percutaneous nephrolithotomy remains the standard treatment for renal calculi larger than 2 cm; however, giant renal pelvic stones are often associated with the need for multiple tracts or staged procedures and carry risks of bleeding, sepsis, and residual fragments. We report the case of a 60-year-old man who presented with chronic left flank pain and burning micturition. Imaging demonstrated preserved renal function with a 5.2-cm solitary left renal pelvic calculus. The patient underwent transperitoneal laparoscopic pyelolithotomy using a 3-port approach, which enabled complete en bloc stone extraction without fragmentation. A double-J stent was placed and the pyelotomy was closed primarily. The postoperative course was uneventful, with complete stone clearance and resolution of symptoms. This case highlights that laparoscopic pyelolithotomy is a safe, feasible, and effective minimally invasive alternative in selected patients with giant solitary renal pelvic stones, providing excellent single-stage stone clearance with minimal morbidity.

## Introduction

Urolithiasis is one of the most common benign diseases of the urinary system, affecting nearly 1%–13% of the global population [1]. The management of renal stones has evolved significantly from open surgery to minimally invasive techniques such as shock wave lithotripsy, retrograde intrarenal surgery, and percutaneous nephrolithotomy (PCNL). Current European Association of Urology (EAU) and American Urological Association guidelines recommend PCNL as the first-line treatment for renal stones ≥2 cm [2]. However, in giant or complex renal calculi, PCNL may be associated with complications such as bleeding, renal parenchymal injury, vascular damage, postoperative sepsis, and incomplete stone clearance, often necessitating multiple tracts or staged procedures [3]. In selected cases, laparoscopic surgery serves as an effective minimally invasive alternative, particularly when endourological approaches are technically challenging or associated with higher morbidity [4]. We report a case of a 5.2-cm left renal pelvic calculus successfully managed with transperitoneal laparoscopic pyelolithotomy, demonstrating its safety, feasibility, and excellent stone clearance.

## Case presentation

**Figure 1:**
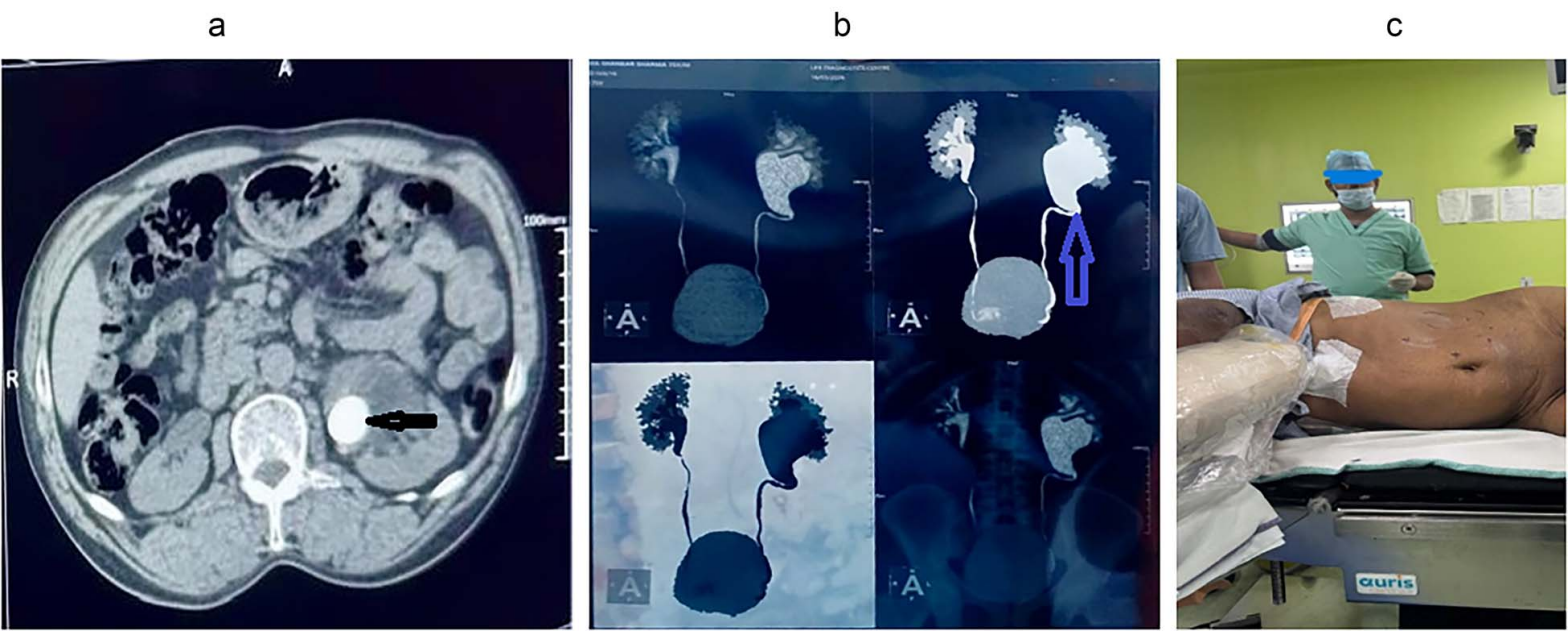
(a) Contrast-enhanced CT urography (coronal section) demonstrating a large radio-opaque calculus measuring 5.2 cm within the left renal pelvis, associated with moderate hydronephrosis (black arrow). (b) CT urography showing preserved bilateral renal excretory function, with contrast outlining the giant calculus occupying the extrarenal pelvis of the left kidney (blue arrow). (c) Patient positioned in a modified left lateral decubitus position, optimized for the transperitoneal laparoscopic approach.

A male patient in his 60s presented to our surgical outpatient department with a 1-year history of intermittent dull aching left flank pain associated with burning micturition, partially relieved by oral analgesics. There was no history of fever, chills, gross hematuria, pyuria, spontaneous stone passage, decreased urine output, or lower urinary tract obstructive symptoms. Routine hematological and biochemical investigations were normal, and renal function was preserved with a serum creatinine level of 0.70 mg/dl. Ultrasonography of the kidney, ureter, and bladder revealed left-sided hydronephrosis with a large renal pelvic calculus. Further evaluation with non-contrast and contrast-enhanced computed tomography (CT) urography demonstrated a large radio-opaque calculus measuring 5.2 cm in the left renal pelvis, with preserved bilateral renal excretory function (Fig. 1a and b).

After detailed counseling regarding the available surgical options, informed consent was obtained for transperitoneal laparoscopic pyelolithotomy (LP). Under general anesthesia, the patient was placed in a modified left lateral decubitus position without elevation of the kidney bridge (Fig. 1c). A transperitoneal laparoscopic approach was employed. A 10 mm camera port was placed 2 cm lateral and superior to the umbilicus. A 5 mm working port was inserted in the left subcostal midclavicular line, and another working port was placed at the junction of the medial two-thirds and lateral one-third of the spino-umbilical line in the midclavicular line.

**Figure 2:**
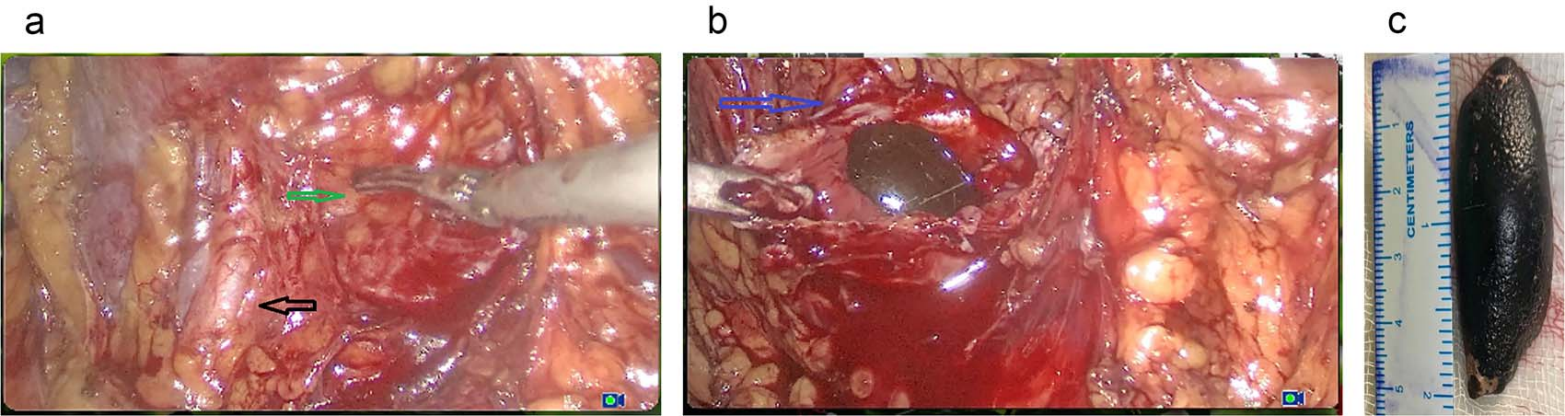
(a) Laparoscopic view of the dissected retroperitoneum demonstrating the left renal vein, renal artery (black arrow), and the markedly dilated extrarenal pelvis containing the calculus (green arrow). (b) Intraoperative image showing the U-shaped pyelotomy incision (blue arrow) made directly over the extrarenal pelvis to safely expose the giant calculus. (c) Intact en bloc extraction of the 5.2 cm giant renal pelvic calculus through the pyelotomy incision.

The peritoneum was incised laterally, and the bowel was reflected medially to expose the retroperitoneum. Gerota’s fascia was opened to identify the upper ureter and dilated renal pelvis. The renal vessels were carefully dissected and preserved (Fig. 2a). A U-shaped pyelotomy incision was made over the extrarenal pelvis (Fig. 2b), and the 5.2 cm renal pelvic stone was extracted intact without fragmentation (Fig. 2c).

A 6 Fr, 26 cm Double-J (DJ) stent was then placed antegradely over a guidewire into the bladder. The pyelotomy was closed primarily over the stent using continuous 4-0 Vicryl sutures to achieve a watertight closure. The specimen was retrieved in an endobag, hemostasis was secured, and the port sites were closed routinely.

## Discussion

With advances in diagnostic imaging and minimally invasive techniques, the incidence of giant renal stones weighing >100 g has markedly declined in modern urological practice. Nevertheless, massive renal calculi continue to pose significant clinical challenges and may lead to recurrent infection, obstruction, and eventual renal failure if left untreated. Nephrolithiasis is more common in males, although staghorn calculi occur more frequently in females. Historically, 49%–68% of staghorn stones were composed of struvite; however, recent studies suggest an increasing prevalence of calcium phosphate stones, highlighting the role of underlying metabolic stone disease [5].

PCNL remains the standard treatment for large renal stones. However, stones larger than 5 cm present considerable technical difficulty and often require multiple access tracts, increasing the risk of renal parenchymal bleeding, arteriovenous fistula formation, and postoperative systemic inflammatory response syndrome [6]. Additionally, stone-free rates decline with increasing stone burden, frequently necessitating staged procedures. In selected patients, LP serves as an effective minimally invasive alternative.

In the present case, the transperitoneal approach provided excellent visualization of the renal hilum and surrounding anatomy. LP enabled intact en bloc stone extraction, ensuring immediate stone clearance without residual fragments [7]. Unlike PCNL, LP avoids direct renal parenchymal puncture, thereby preserving nephron mass and reducing the risk of significant intraoperative or postoperative bleeding [8, 9]. The EAU guidelines also recognize laparoscopy as a valuable option in situations where PCNL may be technically difficult or less effective, such as giant solitary extrarenal pelvic stones, concomitant ureteropelvic junction obstruction, or ectopic kidneys [2, 4]. In this case, the transperitoneal route offered a wider working space for intracorporeal manipulation and suturing. The U-shaped pyelotomy facilitated atraumatic stone extraction, while primary closure over a DJ stent ensured a watertight repair and minimized the risk of postoperative urinary leakage.

## Conclusion

LP is a safe, effective, and minimally invasive option for the management of giant renal pelvic stones in selected patients. In cases with a large solitary stone in an extrarenal pelvis, it offers advantages over PCNL by avoiding renal parenchymal injury, reducing bleeding, and achieving complete stone clearance in a single procedure. In experienced hands, it remains a valuable alternative for the treatment of large renal calculi.

## Data Availability

The data underlying this article will be shared on reasonable request to the corresponding author.
